# Self-assembled bionanostructures: proteins following the lead of DNA nanostructures

**DOI:** 10.1186/1477-3155-12-4

**Published:** 2014-02-03

**Authors:** Helena Gradišar, Roman Jerala

**Affiliations:** 1 Department of Biotechnology, National Institute of Chemistry, Ljubljana, Slovenia; 2 Excellent NMR – Future Innovation for Sustainable Technologies, Centre of Excellence, Ljubljana, Slovenia

**Keywords:** Self-assembly, Protein nanostructures, DNA nanostructures, Protein origami

## Abstract

Natural polymers are able to self-assemble into versatile nanostructures based on the information encoded into their primary structure. The structural richness of biopolymer-based nanostructures depends on the information content of building blocks and the available biological machinery to assemble and decode polymers with a defined sequence. Natural polypeptides comprise 20 amino acids with very different properties in comparison to only 4 structurally similar nucleotides, building elements of nucleic acids. Nevertheless the ease of synthesizing polynucleotides with selected sequence and the ability to encode the nanostructural assembly based on the two specific nucleotide pairs underlay the development of techniques to self-assemble almost any selected three-dimensional nanostructure from polynucleotides. Despite more complex design rules, peptides were successfully used to assemble symmetric nanostructures, such as fibrils and spheres. While earlier designed protein-based nanostructures used linked natural oligomerizing domains, recent design of new oligomerizing interaction surfaces and introduction of the platform for topologically designed protein fold may enable polypeptide-based design to follow the track of DNA nanostructures. The advantages of protein-based nanostructures, such as the functional versatility and cost effective and sustainable production methods provide strong incentive for further development in this direction.

## Introduction

The versatility of biopolymers can be used to rationally design new molecules and assemblies with structures and functionalities unseen in nature. The ability of biopolymers to self-assemble into complex shapes and structures defined at the nanometer scale, and our competence of sustainable large-scale production using cell factories makes them highly desirable for diverse technological applications. In the rapidly-growing research area of modern nanobiotechnology the natural components polypeptides and nucleic acids have been employed as building blocks for the assembling of new designed nanostructures and nanomaterials. Bionanotechnologists have in the last decades achieved important advances in protein-based and particularly DNA-based responsive nanostructures, which can now be designed to self-assemble into almost any selected shape.

Molecular self-assembly as the main organizing principle of biological systems is also a widely applied strategy in the nanotechnology as the driving force for the assembly of artificial nanostructures. In self-assembly the final structure is encoded by interactions of its building elements defined by their properties and the order of building blocks within the linear polymer. The shapes and functions of both, DNA- and protein-based nanostructures are encoded by the sequence of their constituents, nucleotides and amino acids. Additionally, the architecture of both type of the nanostructures can be affected also by the environmental factors, such as solvent, pH, temperature and building blocks concentration.

DNA nanostructures are based on the Watson-Crick nucleic base complementarity. There are only two different base pairs based on a specific pairwise interaction, where stacking with neighboring pairs underlies the formation of stable double-helical domains that serve as the nanostructural building blocks. Some of the most spectacular examples of the potentials of nanobiotechnology have been demonstrated by DNA-based nanostructures. In the nature the primary function of nucleic acids are the storage, processing and mediation of genetic information; however natural structures such as aptameres, telomeres and partially the ribosome as one of the key and most complex nanodevices are formed by nucleic acids assembled into 3D structures. The relevance of the physiological role of nucleic acids that perform their function in form of self-assembled noncoding RNA transcripts is still unknown. On the other hand artificial rationally designed DNA nanostructures, which utilize a narrower subset of interactions from aptameres, can adopt a huge diversity of 2D or 3D shapes [1-5].

**Figure 1:**
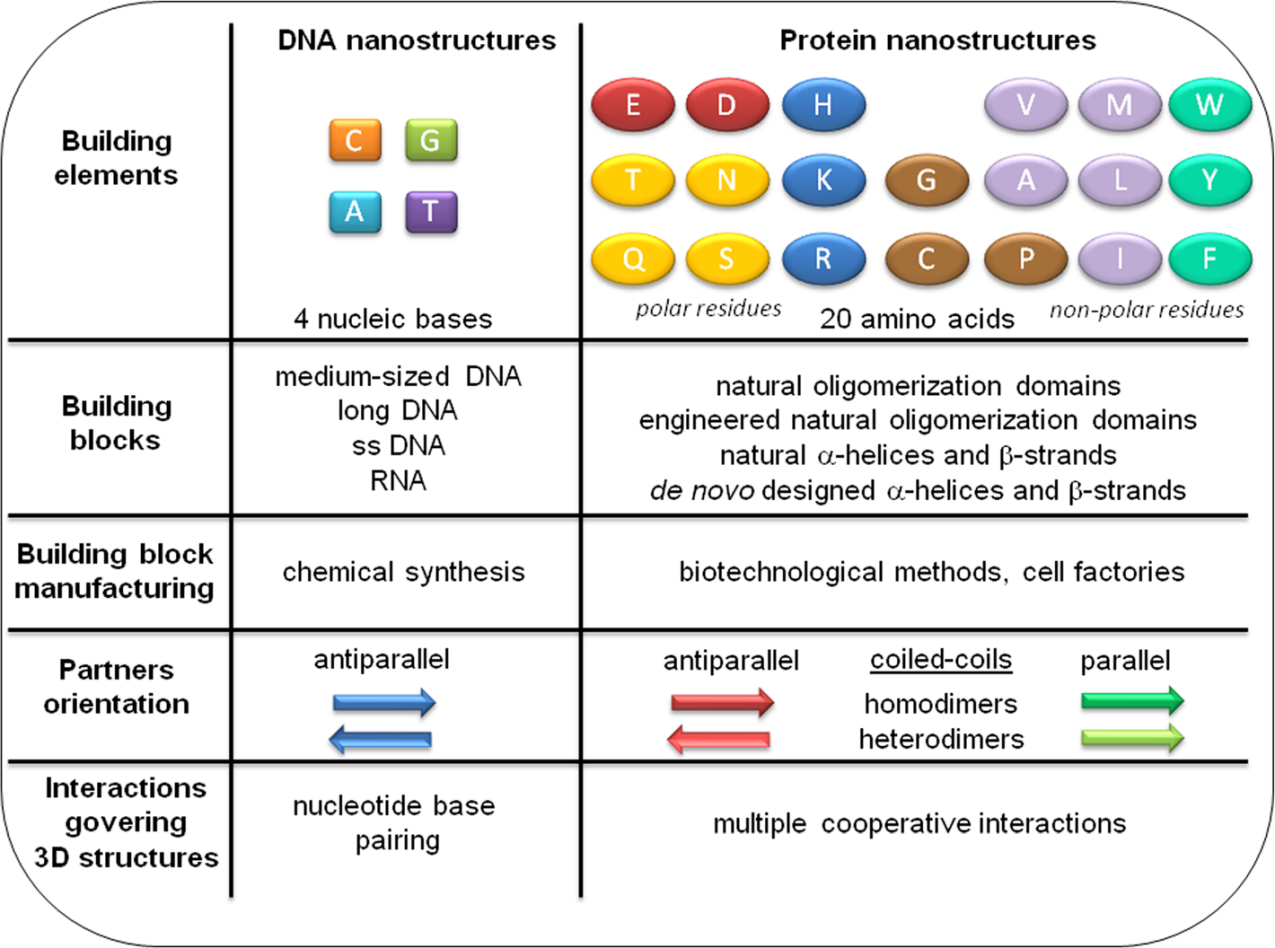
**Some features of self-assembled DNA- and protein nanostructures.** Natural proteins comprise 20 amino acid residues with diverse properties in comparison to only 4 structurally similar nucleotides, building elements of nucleic acids. The advantages of protein nanostructures include also cheaper manufacturing of building blocks, as well as the multiple cooperative interactions that govering protein nanostructures.

In contrast to designed DNA nanostructures, the rational design of protein nanostructures is much more complicated due to the complex cooperative interactions between amino acids stabilizing the fold of native proteins. The comparison of some features of self-assembled DNA- and protein nanostructures is presented in Figure 1. Structural folding of most natural proteins still cannot be easily predicted from their primary structure due to contribution of many cooperative and long-range interactions between amino acids, therefore *de novo* design of completely new protein folds is even more challenging.

However, a significant progress has been recently achieved in the development of strategies for building artificial self-assembled bionanostructures, and a range of both, DNA- and protein nanostructures rapidly increased in last two decades. In this review we mainly focus on protein-based nanostructure strategies, while DNA nanotechnology has been discussed in detail in many recent reviews [6-12].

## Designed DNA nanostructures

In 1982, Seeman proposed to use DNA as the structural material for the bottom-up self-assembly [13] and he is accepted as the founder of the field of DNA nanotechnology. Since then, DNA-based self-assembly achieved spectacular results relying on the base-pairing specificity of nucleotides, using DNA synthesis technology, computer based design and, above all, imaginative design. Over the last three decades self-assembled DNA nanostructures have been extensively studied and several different approaches for building DNA nanostructures have been developed. Self-assembled DNA nanostructures range from 3D structures with a well-defined shape [2,4,14-17] to a variety of complex dynamic DNA devices [8,18-20]. This avenue of research also spawned DNA computing [21,22] and design of dynamic devices [8,23,24], which are however beyond the scope of this review.

**Figure 2:**
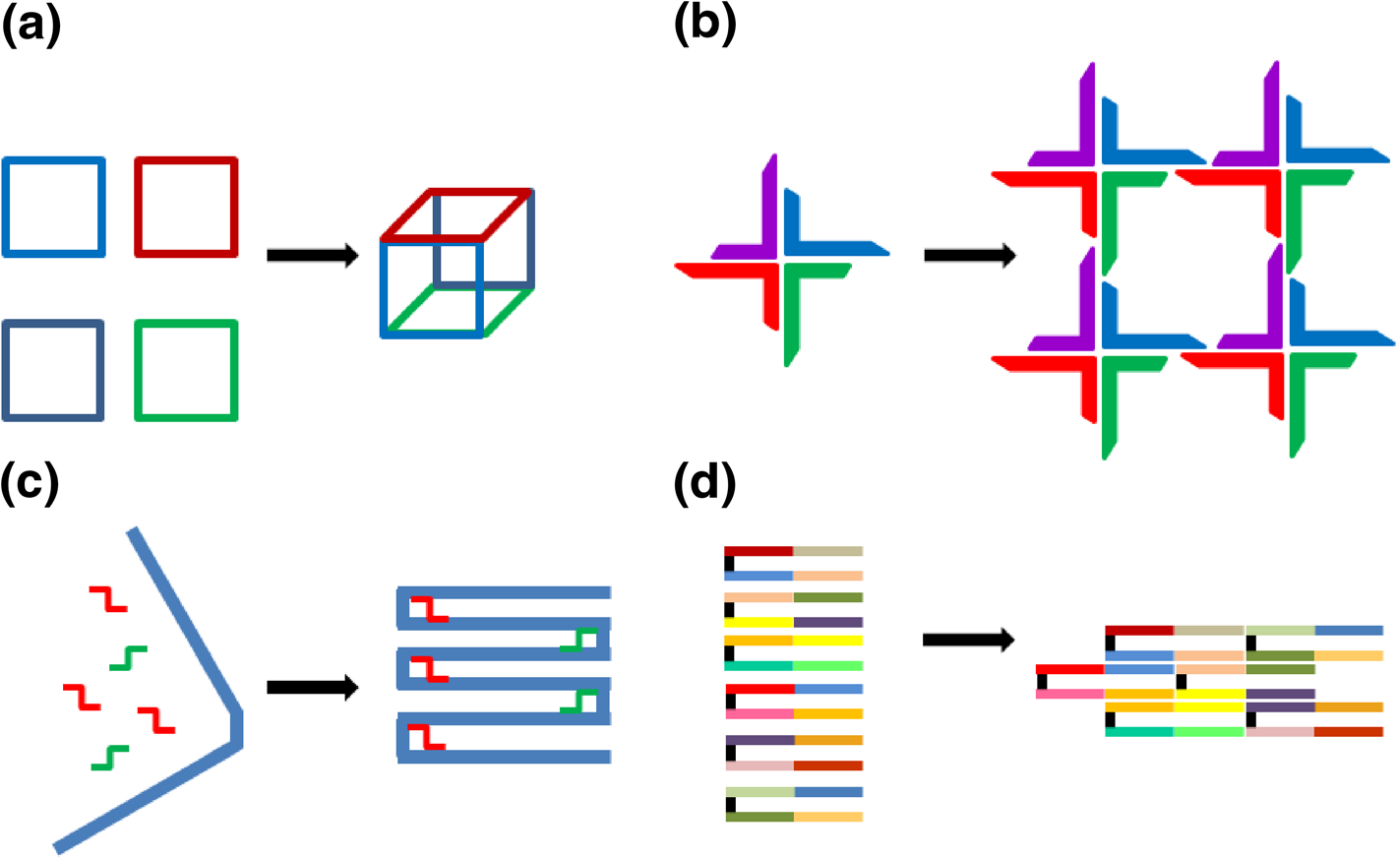
**Different approaches for building DNA nanostructures.** The design of DNA nanostructure is based on the assembly of several medium-sized oligonucleotides that form either **(a)** a finite sized nanostructure or **(b)** assembled building blocks that further oligomerize into a finite sized nanostructure. **(c)** DNA nanostructure can be assembled from a single long DNA scaffold (blue) and short oligonucleotides (red, green) that hold the scaffold in place. **(d)** 2D and 3D nanostructures can be constructed by short DNA strands, DNA bricks.

DNA self-assembly is a robust and flexible biomimetic strategy for molecular construction that is directed by the information embodied in the nucleotide sequence. Development of DNA nanostructures encompasses several different approaches (Figure 2), where the design of nanostructures is based on the assembly of:

– several medium-sized DNA (few 10–100 nucleotides) oligonucleotides that form finite sized nanostructures [14];

– several medium-sized DNA oligonucleotides that assemble into building blocks that further oligomerize into finite sized structures such as different polyhedra or into lattices [3,25];

– single long DNA scaffold (e.g. encompassing several 1000 nucleotides from the single stranded DNA phage) that is shaped into selected structure by the addition of short oligonucleotide clamps a.k.a. *DNA origami technique*, invented by Paul Rothemund [26]. This approach can result in complex 2D or 3D shapes such as molecular raster images, box, sphere etc. [27-30];

– large number of short DNA bricks (32 or 42 nucleotide long strands that form U-shaped brick) that fill the 2D plane or 3D space, where the selected structure is formed by the omission of appropriate DNA bricks from the assembly mixture. Almost any 2D or 3D shape can be formed by this approach [15,31].

An important advantage of DNA-based nanostructures is that it is possible to address the selected positions within the 2D or 3D nanostructures at approximately 5 nm resolution and introduce oligonucleotides with selected functionalities, such as different organic compounds, fluorophores, metal binding groups, proteins etc. into those positions, thereby functionalizing DNA nanostructures [9,32-36].

RNA has the distinct advantage that ssRNA could easily be produced in vivo in order to promote the self-assembly. This property was used to prepare RNA-based scaffolds with attached sites for functional proteins fused to specific sequence RNA binding domains. While those in vivo assembled structures were not well characterized, the scaffold strongly enhanced the reaction yield [37] similar to the DNA-based scaffolded enzymes, where the arrangement of enzymes had been linear [38]. It is hoped that this in vivo approach will be further developed for in vivo applications. ssDNA could also be produced in vivo, demonstrated by the self-assembly of a tetrahedron [39]. Isothermal DNA nanostructure assembly strategy has been developed that could further facilitate future DNA self-assembly in vivo [40].

DNA nanostructures were used to make devices that were functional in the cellular milieu; e.g. drug delivery container that encapsulates cargo, such as therapeutic antibodies, while opening of the container could be controlled by binding of the trigger signals to the aptamer lock that regulates opening of the container only if the triggering signals for both of the two locks are present [41]. DNA origami seems to be stable in vivo indicating that it is relatively protected against nucleases. There are also reports on the use of DNA nanostructures as the constituents of vaccines [42-44]. However real applications of DNA nanostructures are at the moment quite rare and essentially all DNA nanostructures are prepared by chemical synthesis, which limits the technological applications due to the cost and scale of production.

## Protein nanostructures

Proteins provide masterful examples of complex self-assembling nanostructures with properties and functionalities beyond the reach of any human-made materials. It is estimated that there are only few thousand different protein folds in nature, and recently the number of new determined protein fold basically trickled to a halt despite determination of tens of thousands of new protein structures each year. So far folds of only few small protein domains can be accurately predicted [45-48] and design of completely new folds without resemblance to any of the existing native folds represents even a greater challenge [49].

Larger natural proteins have evolved through combinations of several smaller independently folding domains. Protein oligomerization based on the symmetric oligomerization domains is an important source of suprastructured proteins [50]. Existing protein oligomerization domains have been recognized as suitable building blocks for the predictable bottom-up design of artificial protein nanostructures. Strategies that used modified natural domains, or genetically or chemically linked secondary structure elements for self-assembling, and resulted in formation of symmetric intermolecular protein assemblies, lattices and heterogeneous cage-like assemblies, are described in reviews [51-53]. Recently we presented a new approach where a single polypeptide chain composed of concatenated coiled-coil-forming peptides self-assembled into a new topological fold, asymmetric tetrahedron-like cage, which is defined and stabilized by the specific pairing of the coiled-coil-forming segments arranged in a precisely defined order rather than cooperative packing of hydrophobic protein core [54].

### Assemblies based on linked natural protein oligomerizing domains

**Figure 3:**
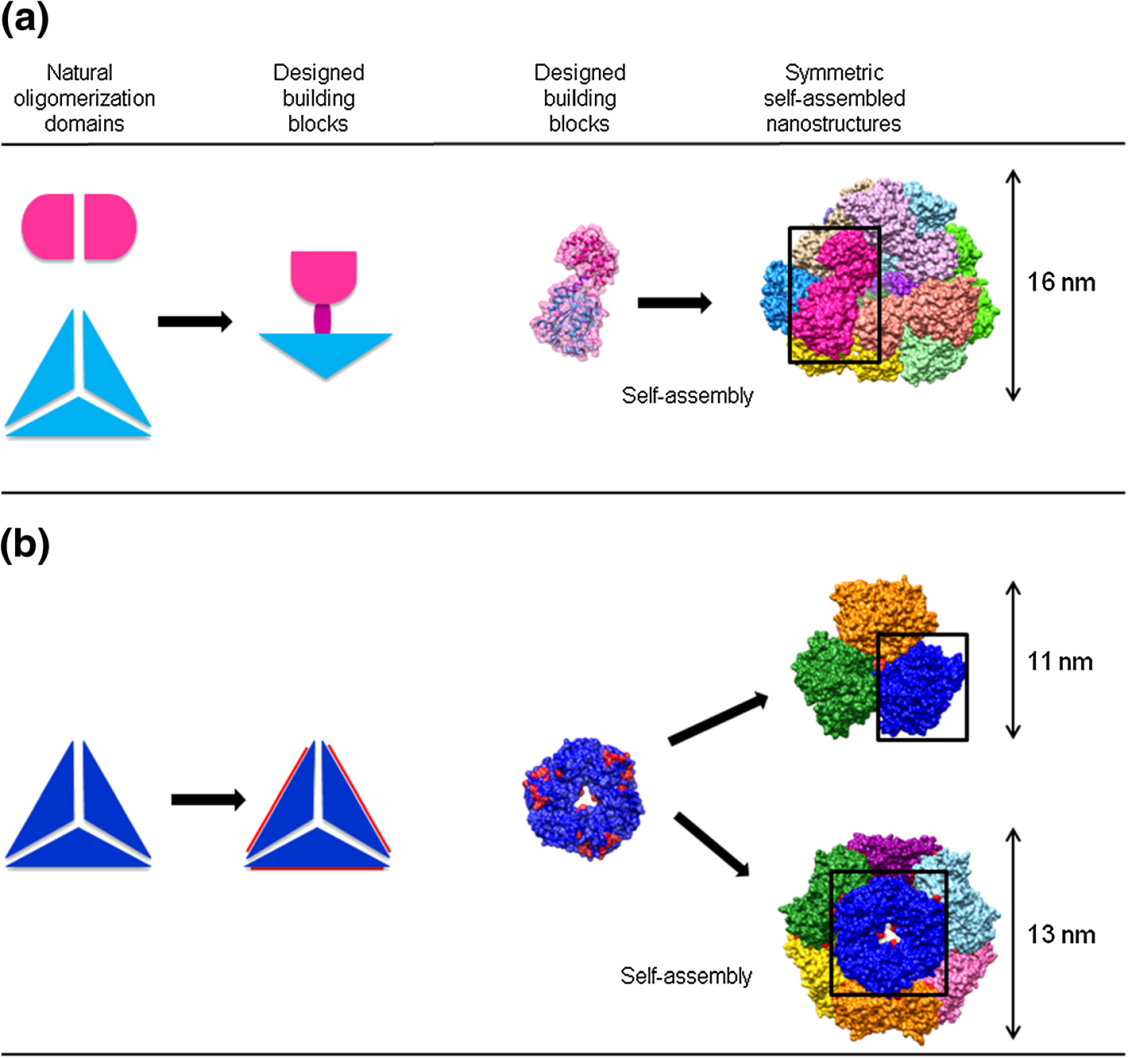
**Design strategies for symmetric domain-based intermolecular protein assemblies. (a)** Fusion of natural oligomerizing protein domains. Two different oligomeric protein domains (dimerization domain (pink), trimerization domain (blue)) are genetically fused via helical linker (violet) to obtain a single chain building block which self-assembled into a 12-subunit cage-like structure with tetrahedral shape (4d9j) [56]. **(b)** Novel protein domain interface design. Computational design of additional interaction surfaces (red) on natural trimerization domain (blue) leads to the formation of 12-subunit assembly with tetrahedral - or 24-subunit assembly with octahedral symmetry (4ddf) [62].

The first strategy for the creation of designed protein nanostructures relied on interactions between oligomerizing protein domains which typically comprise 100–200 or more amino acid residues. The domains can self-assemble non-covalently, but specifically into larger superstructures. Attempts in this direction have been pioneered with fusion strategy [55]. Two different oligomerizing domains, one promoting dimerization and another one promoting homo-trimerization were linked by a semi-rigid linker (Figure 3a). Several copies of such a fusion protein were able to self-assemble into symmetric small cage-like but heterogeneous assemblies, or extended fibrils, depending on the length of the helical linker. Recent refinement of the original protein sequence resulted in a homogeneous 12-subunit assembly, confirmed by X-ray crystal structure determination. The structure of this oligomeric nanostructure reveals tetrahedral geometry with 16 nm diameter [56,57].

This approach provides the possibility to create smart bionanomaterials by regulating the assembly and disassembly. Self-assembly of the fusion protein composed of the dimerizing gyrase B domain and trimerization domain can be driven by the addition of a small molecule. The addition of pseudo-dimeric gyrase B ligand, coumermycin, induced formation of hexagonal assemblies and its dissociation by the subsequent addition of a monomeric ligand novobiocin, which competes for binding to the same gyrase B site as the pseudodimeric coumermycin [58].

The extended fusion strategy circumvented the problem of connecting two oligomerization domains in a fixed relative orientation which assured well-ordered self-assembled protein nanostructures [59]. They showed that fusion protein can be made by selecting two or more connections between the adjacent oligomers if the two domains are joined along an axis of symmetry that both oligomerization domains share. However this symmetry-matching fusion protein strategy successfully manufactured linear filaments, two-dimensional lattices and large solid aggregates, but is not suitable for designing defined cage-like structures.

### Engineering new interaction surfaces into native protein domains

In the strategies described above the range of suitable protein domains is limited by restrictions regarding the symmetry axes of the natural domains. A step further towards the design of artificial protein nanostructures was done by engineering domain surfaces for weak non-covalent interactions in the self-assembling processes. The analysis of natural contact interfaces between protein domains disclosed the rules governing domain association. The contacting surfaces should be complementary and predominantly non-polar. The contribution of hydrogen bonds and salt bridges at the contact rim is negligible. Employing these rules it was demonstrated that a given protein can be engineered to form new contact interfaces that produced a number of novel assemblies [60]. Algorithm Rosetta for modeling protein-protein interactions [61] enables *de novo* design of interacting interfaces which can drive the self-assembly of designed proteins into a desired symmetric architecture [46,62]. In a recent study, a computational design of protein nanostructures with atomic level accuracy was described [62]. Protein building blocks, based on natural trimeric protein domains were docked together symmetrically to the target packing arrangements and low-energy protein-protein interaction interfaces were designed between building blocks in order to drive the self-assembly (Figure 3b). The designed proteins assembled into cage-like nanostructures with either tetrahedral or octahedral point group symmetry which was confirmed by crystal structures.

### Modular approach for *de novo* designed protein nanostructures

The strategies employing oligomerizing protein domains for designing new protein structures, described above, are limited to homologues of known native protein folds. The next generation engineering approaches are based on modules that can be considerably smaller than the typical protein domain. The modules comprise interacting *de novo* designed secondary structure elements that are predictably combined with specified partners to form larger assemblies. *De novo* protein design refers to attempts to construct completely new protein sequences for the prescribed structures based on the principles defining the stability and selectivity of building modules; in *de novo* design the polypeptide sequence is selected by the designer.

Modularity and orthogonality are two foundation concepts of *de novo* design and engineering of new protein nanostructures. Instead of optimization of the numerous cooperative interactions that underpin the structures of natural proteins, the use of well-understood structural modules, which could be combined into complex nanostructures, was proposed. α-helices and β-strands represent attractive protein folding motifs to serve as building blocks for well-ordered and defined nanostructures with complex architecture [63-67].

The most studied module for building self-assembled protein nanostructures are interacting helical peptides and particularly coiled-coils. They are ubiquitous facilitators of inter- and intramolecular protein-protein interactions and comprise two or more intertwined α-helices that are encoded by the characteristic heptad sequence repeat, where residues are labeled with *abcdefg*. The non-covalent interactions that drive the formation of coiled-coils are the hydrophobic effects between amino acids at positions *a* and *d* that form a hydrophobic core of coiled-coil, and the electrostatic inteactions between the opposite charged residues at positions *e* and *g*. The rules governing coiled-coil formation, their oligomerization state and interaction partner specificity have been considerably established over the last decades [68,69]. On the basis of those rules sets of orthogonal designed coiled-coils as the toolkit for the designed protein assemblies were developed [70-75]. Engineered coiled-coil polypeptides have been used to assemble different nanomaterials: nanofibres [76,77], membranes [78], nanotubes [79], nanostructured films [80], spherical structures [81], responsive hydrogels [82,83], spheres [84] etc. Homogeneous nanoparticles with regular polyhedral symmetry, about 16 nm in diameter, were prepared from single type of polypeptide chains where the two coiled-coil modules with different oligomerization states were joined by a short linker [85]. In another study two oligomerizing coiled-coil peptides were tethered via disulphide bond close to their center. The self-assembled molecules spontaneously curved into the spherical cage-like particles, with a hexagonal-pattern of the cage surface and about 100 nm in diameter [84]. Another example are discrete circular nanostructures of defined stoichiometry; trimers or tetramers of < 10 nm were observed when linker between two coiled-coil-forming segments comprising 6–10 residues. Larger colloidal-scale assemblies as well as flexible fibers were formed when shorter linkers limited flexibility between peptides [86].

### Designed topological protein folds based on interacting coiled-coil modules

Recent innovative approach to construct new engineered self-assembled protein nanostructures is based on the concatenated interacting dimerizing modules, comprise up to 45 amino acid residues [54]. The tetrahedral nanostructure was built from only single polypeptide chain; this strategy may appropriately be called *designed protein origami* as opposed to native protein structures that fold into a defined 3D structure from a single chain.

Rather than folding the structure based on the interactions between residues in the hydrophobic core as for the native proteins, the modular topological design is based on pairwise interactions between concatenated secondary structure elements (coiled-coil-forming segments), whose folding and orthogonality is engineered independently. Orthogonality of used coiled-coil building modules ensures that each segment preferentially binds to its designated partner segment within the same polypeptide chain. The final topology is defined by the sequential order of coiled-coil segments. The topological fold comprises a cavity bounded by coiled-coil dimers as the edges of the polyhedron. This type of modular self-assembly therefore in many aspects resembles the principles of DNA nanostructures [2,3,26], where polyhedra had been constructed based on the complementary DNA segments.

**Figure 4:**
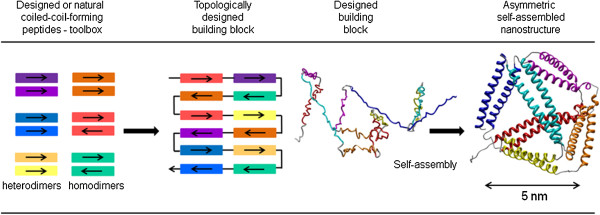
**Protein origami: modular topological design of protein structure from a single polypeptide chain.** A toolbox for constructing tetrahedron-like cage comprised of six orthogonal pairs of coiled-coil-forming peptides, two antiparallel- and four parallel dimers (orientation is denoted by arrow). Twelve peptides were concatenated in a defined order, separated by the tetrapeptide linker. The single polypeptide chain served as a building block that self-assembled into monomeric and asymmetric tetrahedron-like nanostructure [54].

According to this approach long range non-covalent interactions occur between coiled-coil-forming segments, which dimerize independently of the other segments. The coiled-coil-forming segments are concatenated into a precisely defined order with intervening flexible linkers between each segment, to provide the hinge-like flexibility. In the case of a monomeric tetrahedron, which was constructed to demonstrate the principle, the polypeptide chain is composed of 12 designed coiled-coil dimer- forming segments, each forming an orthogonal coiled-coil dimer with its partner segment within the same polypeptide chain (Figure 4). In this way it forms 6 edges of a tetrahedron, while the flexible linkers were positioned at vertices. The polypeptide was produced in the recombinant form in *E. coli* and self-assembled by a slow dialysis or temperature annealing into tetrahedral structure, whose edges measure around 5 nm. This direction opens an exciting perspective for the creation of additional entirely new protein folds. The principle of protein assembly can benefit significantly by the application of a mathematical topology theory, which can be used to analyze the number of theoretical solutions and may be in the future applied to optimize the kinetics of the assembly [87]. The results of protein nanocage engineering show that modular design can be used for complex structures, with the potential for applications biocatalysis, targeted drug delivery, vaccination, etc. [88].

## Conclusions and future prospects

The recent successes in the design of new bionanostructures based on DNA and protein demonstrates the potentials of this approach to engineer new functional nanostructures.

While DNA-based nanostructures are clearly ahead of the designed protein nanostructures in terms of the complexity of the designed structures so far they lacked tangible applications. Although it has been demonstrated that DNA-based nanostructures are functional in organisms, use of in vivo produced and assembled nucleic acid-based nanostructures would represent an important step ahead both for the production cost and new biological applications. Functionalization of nucleic acids could combine structural design with precisely addressed functionalities. However, proteins adopt much larger conformational variability than nucleic acids and provide more versatile functionality. *De novo* design of protein nanostructures has been limited to small number of application cases which predominatly utilizing repurposed natural protein domains. Nevertheless the design of protein assemblies has matured beyond the proof of principles and is ready to face more complex challenges. New emerging paradigms such as the topological protein folds open completely new avenues that seem not to have been adopted or perhaps even tested by nature. Future developments will demonstrate the potentials of different strategies, or their combinations, with respect to the precise engineering of nanostructures and the theoretical limitations of different platforms. The next stage will need to focus on application development. The potentials are numerous, from targeted drug and biomolecule delivery, vaccine design, tissue engineering, senzors design, biocatalysis to bionanomaterials science. The interdisciplinary approach of synthetic biology, combining structural biology, molecular biology, mathematics, engineering and many other disciplines, have the potential to join forces in this exciting opportunity.

## Abbreviations

2D: Two-dimensional; 3D: Three-dimensional.

## Competing interests

The authors declare that they have no competing interests.

## Authors’ contributions

HG and RJ participated equally in writing this manuscript. Both authors read and approved the final manuscript.
